# Pathology of bladder cancer among diabetic patients undergoing radical cystectomy with a history of pioglitazone (Actos) use

**DOI:** 10.1186/1471-2490-14-10

**Published:** 2014-01-25

**Authors:** Victoriano Romero, Charles Peyton, Ian Gray, Ashok Hemal, Ryan Terlecki

**Affiliations:** 1 Department of Urology, Wake Forest Baptist Medical Center, 27157, Winston Salem, NC, USA

**Keywords:** Pioglitazone, Bladder cancer, Diabetes, Tumor stage

## Abstract

**Background:**

Prospective studies suggested an association between pioglitazone (Actos) use and the development of bladder cancer. Cancer pathology among pioglitazone users has not been characterized. We chose to compare the surgical pathology among diabetic users and non-users, as well as non-diabetic patients who underwent radical cystectomy for bladder cancer.

**Methods:**

Our single-center, prospectively-maintained bladder cancer database was reviewed. Patient demographics, surgical pathology, and outcomes were evaluated. Information regarding diabetic history and use of pioglitazone was determined from chart analysis and patient interview.

**Results:**

From April 2005 to October 2011, 204 patients undergoing radical cystectomy were identified. Of these, 33 (16.2%) were diabetic and 171 (83.8%) had no history of diabetes. Among diabetic patients, 9 (27.3%) had a history of pioglitazone use. Median duration of therapy was 14 (6–120) months. Pathology in non-diabetic patients was T1 in 17 (9.9%), T2 in 38 (22.2%), T3 in 44 (25.7%), and T4 in 31 (18.1%). Pathology among diabetic non-users was T1 in 1 (4.2%), T2 in 7 (29.2%), T3 in 7 (29.2%), and T4 in 4 (16.7%). Pathologic stage among diabetic users was T1 in 1 (11.1%), T2 in 3 (33.3%), T3 in 3 (33.3%), and T4 in 1 (11.1%). Lymph node involvement in non-diabetics, diabetic non-users, and diabetic users was 25.7%, 33.3%, and 33.3%, respectively. Cancer-specific death was seen in 60.3% of non-diabetics, 58.3% of diabetic non-users, and 75% of diabetic users.

**Conclusions:**

Diabetics have similar stage distribution regardless of pioglitazone use. Lymph node metastases rates and cancer specific death were similar across all groups. Additional studies will serve to better characterize this relationship.

## Background

Peroxisome-proliferator-activator-receptor (PPAR)-γ is a ligand activated nuclear transcription factor that is expressed in normal urothelium, but, more importantly, over-expressed in bladder tumors [1-3]. The three isoforms of PPARs (α, γ, and δ) are important targets in regulating normal adipose proliferation and insulin sensitivity [4,5]. As such, PPAR agonists – thiazolidinediones (TZD) – have become a common method of combating insulin resistance. However, preclinical investigations have linked pioglitazone (Actos), a PPAR-γ agonist thiazolidinedione, to developing bladder tumors in animal studies [6-8].

In 2005, a large European randomized control trial (PROactive) evaluating the effects of piolglitazone in prevention of macrovascular events noted a non-significant increase in the number of bladder cancers among piolglitazone users versus those taking a placebo (14 out of 2605 vs 6 out of 2633, respectively) [9]. Given the aforementioned preclinical studies and this clinical observation, the United States Federal Drug Administration [10] launched a 10-year cohort study to evaluate the association between pioglitazone and bladder cancer [11]. A midterm evaluation of the enrolled patients revealed no significant association between short-term pioglitazone use and bladder cancer; however, pioglitazone use for more that 2 years was weakly associated with an increased risk of developing bladder cancer [11]. Another cohort study in France demonstrated a significant dose-dependent association between pioglitizone use and bladder cancer, sparking removal from the French market and the US Food and Drug Administration warning for using pioglitazone in patients with a current or previous bladder cancer [10,12]. Furthermore, a recent meta-analysis of the link between pioglitazone and bladder cancer suggests, “cautious interpretation” of the limited available data and encourages further investigation [13].

Although there has been considerable interest in the incidence of bladder cancer after pioglitazone use, a PubMed search of the peer-reviewed literature fails to reveal any data characterizing surgical pathologic staging among bladder cancer patients with a history of pioglitazone use. To better understand the characteristics of bladder cancers that may develop following pioglitazone use, we sought to evaluate tumor staging among non-diabetics and diabetic users and non-users of pioglitazone undergoing cystectomy at our institution.

## Methods

We reviewed our Wake Forest University Health Sciences Institutional Review Board approved, prospectively maintained database of patients having undergone radical cystectomy for bladder cancer from April 2005 – October 2011. Patient demographics, medical co-morbidities, medication use, operative details, surgical pathology, and outcomes were evaluated. Information regarding diabetic history and use of pioglitazone was gathered from chart analysis and telephone interview.

Statistical analysis was conducted using GraphPad Prism software, version 4.0. Student t-tests were used to compare group characteristics/demographics as mean ± SEM. Multiple Mann–Whitney U signed rank tests (ie: Kruskal-Wallis tests) were used to compare stage distribution between groups (i.e., non-diabetics, diabetic users of pioglitazone, and diabetic non-users). A p < 0.05 was considered significant. Only statistically significant p-values were included in data tables.

## Results

A total of 204 patients undergoing radical cystectomy by three different surgeons were identified. Of these, 33 (16.2%) had insulin-dependent diabetes mellitus and 171 (83.8%) had no history of diabetes. Among diabetic patients, 9 (27.3%) had a history of pioglitazone use, and all were white males. Twenty-four (72.7%) of diabetics in this study were not exposed to Actos. Patient demographics and general co-morbidities are summarized in Table 1. Not surprisingly, the presence of cardiovascular disease was significantly higher among diabetic patients (57.6% versus 26.9%, p < 0.01), as was BMI (29.31 versus 26.3, p < 0.05). Median duration of pioglitazone therapy was 14 months (range 6–120).

**Table 1:** Demographics and medical co-morbidities of bladder cancer patients undergoing cystectomy with only statistically significant p-values shown

	**All patients**		**p-value**	**Diabetic subgroups**		**p-value**
	**Non diabetic**	**Diabetic**		**Pioglitazone non-users**	**Pioglitazone users**	
N	171	33		24	9	
Age (years ± SEM)	67.7	67.2		66.6 ± 1.5	68.7 ± 1.8	
Sex (male)	68.4%	88%	0.009^a^	88%	100%	
Race/ethnicity: No. (%)						
White	157 (91.8)	31 (94)		22 (92)	9 (100)	
Black	13 (7.6)	1 (3)		1 (4)	-	
Asian	1 (0.6)	-		-	-	
Hispanic	-	1 (3)		1 (4)	-	
Smoking	71 (41.5)	12 (36.4)		5 (20.8)	7 (77.8)	0.012^a^
CV disease	46 (26.9)	19 (57.6)	0.01^a^	13 (54.2)	6 (66.7)	
Pulmonary disease	26 (15.2)	7 (21.2)		5 (20.8)	6 (66.7)	
Creatinine (mg/dL)	1.17	1.21		1.2 ± 0.07	1.23 ± 0.11	
BMI	26.3	29.31	0.005^a^	29.2 ± 1.24	29.5 ± 2.3	

^a^student *t*-test.

Tumor stage distribution is shown in Table 2. There was a wide distribution of tumor stage within all groups, with the majority of bladder cancers ranging from T2 – T4. Surgical pathologic stage distribution among all diabetics (eg: combined pioglitazone users and non-users) tended to be more advanced; however this finding was not statistically significant. Muscle invasive disease was not significantly different between non-diabetics, diabetic non-users, and diabetic Actos users (66.1%, 75%, and 77.8% respectively, p = 0.51). Lymph node positivity was not significantly different between non-diabetics, diabetic non-users, and diabetic Actos users (25.7%, 33.3%, and 33.3% respectively, p = 0.71). Cancer specific death was not significantly different between non-diabetics, diabetic non-users, and diabetic Actos users (22.2%, 29.2%, and 33.3% respectively, p = 0.37).

**Table 2:** Bladder cancer stage distribution among non-diabetics and diabetic non-users and users of pioglitazone (Actos)

	**All patients**		**Diabetic subgroups**	
	**Non diabetic**	**Diabetic**	**Pioglitazone non-users**	**Pioglitazone users**
**Stage:** No. (%)				
T0	18 (10.5)	3 (9.1)	2 (8.3)	1 (11.1)
Ta	7 (4.1)	1 (3)	1 (4.2)	-
Tis	16 (9.3)	2 (6)	2 (8.3)	-
T1	17 (9.9)	2 (6)	1 (4.2)	1 (11.1)
T2	38 (22.2)	10 (30.3)	7 (29.2)	3 (33.3)
T3	44 (25.7)	10 (30.3)	7 (29.2)	3 (33.3)
T4	31 (18.1)	5 (15.2)	4 (16.7)	1 (11.1)
**Non muscle invasive**	58 (34)	8 (24.2)	6 (25)	2 (22.2)
**Muscle invasive**	113 (66)	25 (75.8)	18 (75)	7 (77.8)
**Node positive disease**	44 (25.7)	11 (33.3)	8 (33.3)	3 (33.3)

*DM* diabetes mellitus.

Comparison of groups found no statistically significant difference.

## Discussion

Pioglitazone (Actos^TM^) has long been recognized as an effective medication in the management of diabetes, and has been previously recorded as a best-selling drug [7]. It has been widely used as both monotherapy and as part of a multidrug regimen to address insulin resistance. Although, no warnings regarding the potential for urothelial carcinogenesis were made upon initial release, preclinical rodent studies demonstrated a propensity for development of bladder tumors following treatment with PPAR-γ agonists, a phenomenon potentially lessened by urinary acidification [6]. Additionally, studies on human bladder tumor specimens have demonstrated an increased density of PPAR-γ receptors compared to normal bladder tissue, with increased expression among higher-grade tumors [3]. While preclinical studies demonstrated pro-neoplastic effects of PPAR-γ agonists, this class of medication received a largely favorable safety profile due in part to cardioprotective effects [7,9].

At the conclusion of the PROactive study, pioglitazone was associated with a significant decrease in all cause mortality, non-fatal myocardial infarction, and stroke [9]. Considering only bladder cancer that was diagnosed a year or more after the onset of the study, tumors were seen in six patients in the pioglitazone arm and in three patients in the placebo arm, one of which was later reported to have benign pathology. This finding was not statistically significant, and remained non-significant in 4 years of follow-up [14]. *Hillaire-Buys et al.* performed an updated repeat analysis of the PROactive data excluding one case in the placebo group that actually showed benign pathology, and only then was a statistically significant increase in bladder cancer incidence determined for pioglitazone users [15].

The FDA has commissioned a 10-year epidemiologic study gathered retrospective data on 193,099 diabetic patients and identified 30,173 pioglitazone users, with 90 of these having developed bladder tumors [11]. Although the 5-year midterm analysis found that pioglitazone use for more than 2 years had a weak association with increased cancer risk, there was no statistically significant short-term use risk and no evidence of a tumor stage shift [11]. Additional epidemiologic studies led to suspension of the use of pioglitazone in France, and a warning in Germany to avoid prescriptions for non-users [12]. On June 15, 2011, the USFDA officially issued a similar warning.

Despite the work investigating an association between pioglitazone and the development of bladder cancer, no reports have shown a detailed comparison of pathologic tumor staging and patient outcomes between users of this medication when compared to other patients. Based on a PubMed literature search at the time of manuscript preparation, our study is the first to examine the association of pioglitazone use on pathologic staging of cystectomy specimens, surgical presence of lymph node involvement, or cancer-specific survival. Although our cohort is small, the cancers associated with pioglitazone use in our series were unique to male patients. Preclinical rodent studies have found the same sexual predilection [6]. This may suggest a hormonal or chromosomal factor yet unidentified. Our cohort also demonstrated a higher propensity for muscle invasive disease among pioglitazone users. Although this did not reach statistical significance, perhaps a larger cohort would better elucidate this relationship.

This investigation was limited by the retrospective nature of the database, and small number of pioglitazone users identified. Also, the cumulative dose and dose duration was not fully accounted for, secondary to reliance on retrospective data and recall bias on the part of patients. Moving forward, we are collecting this data on patients and also giving attention to findings such as urine pH. Information on use of diabetic medications that could reduce the incidence of bladder cancer should also be gathered. Metformin, for example, has been shown in some studies to protect against bladder cancer development, and the interaction between metformin and pioglitazone could further define treatment strategies for management of Type II diabetes. Future efforts will also include analysis of those bladder cancer patients not undergoing cystectomy (e.g., TURBT +/- adjuvant therapies). We will also aim to determine PPAR-γ receptor density among cases. Furthermore, drawing data from larger sources (e.g., SEER database) may serve to shed more light on this matter.

## Conclusions

In our experience, diabetics undergoing radical cystectomy have similar stage distribution regardless of pioglitazone use. The rate of lymph node metastases and cancer specific death was similar across all groups. Additional study and further follow-up may better characterize this relationship.

## Consent

Due to the retrospective nature of this study, informed consent was not obtained in accordance with IRB approval.

## Competing interests

The authors declare that they have no competing interests.

## Authors’ contributions

VR carried out database analysis, drafted and edited the manuscript, and performed background research for inclusion in the manuscript. CP performed the retrospective review of electronic medical records, carried out database analysis, edited the manuscript, and performed statistical analysis of acquired data. IG performed the retrospective review of electronic medical records, carried out database analysis. AH was the primary surgeon who generated the data for review, carried out database analysis, and provided editorial commentary during manuscript creation. RT drafted and edited the manuscript, performed background research for inclusion in the manuscript, provided editorial commentary during manuscript submission, and designed the retrospective review. All authors read and approved the final manuscript.

## Pre-publication history

The pre-publication history for this paper can be accessed here:

http://www.biomedcentral.com/1471-2490/14/10/prepub
